# Assessment of the Impact of Antimicrobial Photodynamic Therapy Using a 635 nm Diode Laser and Toluidine Blue on the Susceptibility of Selected Strains of *Candida* and *Staphylococcus aureus*: An In Vitro Study

**DOI:** 10.3390/microorganisms13092126

**Published:** 2025-09-11

**Authors:** Marcin Tkaczyk, Anna Mertas, Anna Kuśka-Kiełbratowska, Jakub Fiegler-Rudol, Elżbieta Bobela, Maria Cisowska, Dariusz Skaba, Rafał Wiench

**Affiliations:** 1 Department of Periodontal Diseases and Oral Mucosa Diseases, Faculty of Medical Sciences in Zabrze, Medical University of Silesia, 40-055 Katowice, Poland; anna.kuska-kielbratowska@sum.edu.pl (A.K.-K.); s88998@365.sum.edu.pl (J.F.-R.); rwiench@sum.edu.pl (R.W.); 2 Department of Microbiology and Immunology, Faculty of Medical Sciences in Zabrze, Medical University of Silesia in Katowice, Jordana 19 Str., 41-808 Zabrze, Poland; amertas@sum.edu.pl (A.M.); ebobela@sum.edu.pl (E.B.); mcisowska@sum.edu.pl (M.C.)

**Keywords:** antifungal therapy, TBO, photosensitizer, diode laser, candidiasis, photodynamic inactivation, oral infections

## Abstract

Yeasts of the genus *Candida* (*C.*) and the bacterium *Staphylococcus aureus* (*S. aureus*) are among the most common pathogens responsible for infections that are difficult to treat, including those resistant to standard therapy. In recent decades, this has become an increasing clinical problem. In response to the limitations of traditional procedures, antimicrobial photodynamic therapy (aPDT), which combines light, a photosensitizer, and oxygen, is gaining growing interest. The aim of this study was to evaluate the in vitro effectiveness of aPDT using a 635 nm diode laser in combination with toluidine blue O (TBO) against *Candida* spp. and *S. aureus*. Reference strains of *C. albicans*, *C. glabrata*, *C. krusei*, and *S. aureus* were subjected to aPDT. In phase I of this study, the optimal TBO incubation time was assessed with constant laser parameters. In phase II, the impact of the physical parameters of the laser, irradiation time, and output power, was analyzed, with the TBO incubation time set based on the phase I results, to evaluate the degree of microbial reduction (CFU/mL). Statistical analyses were then conducted to assess significance. TBO-mediated aPDT significantly reduced microbial viability, depending on incubation time and laser settings. The minimal effective incubation times were 10 min for *Candida* spp. and 5 min for *S. aureus*. The highest pathogen inactivation efficacy was observed at an output power of 400 mW and an irradiation time of 120 s. The use of the photosensitizer or laser alone did not result in significant antimicrobial effects. TBO-mediated aPDT may serve as an effective complement to conventional antimicrobial therapy and, in selected cases (e.g., drug resistance), has the potential to partially or fully replace it. The observed minimal effective incubation times provide a practical baseline, but further statistical comparisons are required to determine whether these durations are truly optimal.

## 1. Introduction

*Candida* spp. cause infections ranging from superficial mucosal or skin lesions to severe, invasive disease [1,2], with incidence rising in recent years [3]. *Candida albicans* remains the most common isolate (~70%) [4], but infections from non-albicans *Candida* (NAC) species are increasing [5], driven by improved diagnostics (chromogenic media, molecular techniques) and the higher virulence and antifungal resistance of NAC strains, which correlate with increased mortality [6,7]. *Staphylococcus aureus* is a Gram-positive, coagulase-positive commensal that also acts as a major pathogen, responsible for hospital- and community-acquired infections including pneumonia, bacteremia, endocarditis, and osteomyelitis [8,9,10,11,12]. Its adaptability and rapid resistance development complicate treatment. Methicillin-resistant *S. aureus* (MRSA) shows resistance to all penicillins and most beta-lactams, with only ceftaroline and ceftobiprole effective. Vancomycin remains the last-line therapy, but VISA and VRSA have emerged [9,13,14]. Resistant infections continue to rise globally, with pan-resistant strains a major concern [15,16], and projections suggest many current treatments may lose effectiveness within 30 years [17]. These challenges have prompted investigation of alternatives such as essential oils, plant extracts, propolis, probiotics, nano-metal colloids, ozone therapy, and antimicrobial photodynamic therapy (aPDT) [18,19,20,21,22]. aPDT combines light of a defined wavelength, a photosensitizer (PS), and oxygen [23,24]. After administration, the PS accumulates in tissues; irradiation then generates singlet oxygen and other ROS, selectively damaging microbial cells [20,21,22,25]. Toluidine blue ortho (TBO), a small, water-soluble cationic PS, binds microbial membranes [21] and is effectively activated in the 600–660 nm range [26]. Because ROS act non-specifically, aPDT can eliminate drug-resistant microorganisms without promoting resistance [27,28]. This study aimed to optimize TBO incubation time for *Candida* spp. and *S. aureus* and evaluate aPDT using a 635 nm diode laser. Prior work has often been limited to single microbial groups, inconsistent parameters, or lacked comparison across multiple *Candida* species and *S. aureus*. Few studies addressed resistant non-albicans *Candida* or mixed suspensions mimicking polymicrobial infections. Here, we systematically assessed TBO-mediated aPDT against *C. albicans*, *C. krusei*, *C. glabrata*, and *S. aureus*, individually and in combination, to establish effective incubation and laser parameters, supporting future standardized protocols and potential clinical use of aPDT against antimicrobial resistance.

## 2. Materials and Methods

The study was carried out at the Microbiological Laboratory of Silesia LabMed, within the Department and Division of Microbiology and Immunology, Faculty of Medical Sciences in Zabrze, Medical University of Silesia in Katowice.

### 2.1. Reference Microbial Strains

Standardized microbial reference strains were obtained from the American Type Culture Collection (ATCC, Manassas, VA, USA). The fungal panel included *C. albicans* (ATCC 10231), *C. glabrata* (ATCC 2001), and *C. krusei* (ATCC 14243), while the bacterial strain was *Staphylococcus aureus* (ATCC 25923). Fungal isolates were subcultured on Sabouraud agar (bioMérieux SA, Marcy l’Étoile, France), and *S. aureus* on Columbia agar with 5% sheep blood (bioMérieux SA). Strains were routinely passaged every 48–72 h at 37 °C according to ATCC protocols to maintain viability and reproducibility. For experiments, 24 h cultures were harvested to prepare suspensions in sterile 0.9% sodium chloride, standardized to 3.0 × 10^8^ CFU/mL (1.0 McFarland). Suspension density was verified with a Densi-La-Meter II (Erba Polska Sp. z o.o., Kraków, Poland).

### 2.2. Photosensitizer and Laser

TBO, the photosensitizing agent employed in this study, was used in the form of FotoSan Agent (CMS Dental, Roslev, Denmark), a clinically applicable gel formulation with a concentration of 0.1 mg/mL (0.01%). It was provided in pre-dosed, single-use 1.2 mL syringes, designed for immediate clinical use without further preparation. Illumination was delivered using a SmartmPro diode laser (Lasotronix, Piaseczno, Poland), emitting light at a wavelength of 635 nm and operating in continuous wave (CW) mode. The laser applicator featured an 8 mm diameter tip, covering an area of approximately 0.5 cm^2^. The emitted beam followed a Gaussian spatial intensity profile, with peak intensity at the center that tapered toward the periphery. The laser’s output power was adjustable between 50 and 400 mW, and irradiation durations ranged from 30 to 120 s. These settings corresponded to energy densities (fluence values) between 1.5 and 48 J/cm^2^.

### 2.3. First Phase of the Study

The First phase of the study included an assessment of the Impact of Optimal TBO Incubation Time in Experimental Groups on the Reduction in Viable Microbial Cells under Constant Laser Parameters. A total of 384 tests were conducted (96 for each tested microorganism), divided into four experimental groups:

1. (L+P+) aPDT group (suspension subjected to both the photosensitizer and laser light) (n = 4).
2. (L−P+) photosensitizer-only group (suspension exposed to PS without laser irradiation) (n = 4).
3. (L+P−) light-only group (suspension exposed to laser light without PS) (n = 4).
4. (L−P−) control group (suspension without exposure to either laser light or PS) (n = 4).

To assess the impact of incubation time on antimicrobial efficacy, TBO pre-irradiation periods of 1, 5, 10, 15, 20, and 30 min were tested. Laser parameters were kept constant (400 mW, 60 s) to isolate the effect of incubation, with outcomes measured as CFU/mL. Assays were performed in sterile black 96-well microtiter plates with lids (Thermo Fisher Scientific, Waltham, MA, USA), using 200 µL microbial suspension per well. To prevent cross-light contamination, only 24 non-adjacent wells were used per plate. Wells in the (L+P+) and (L−P+) groups received 50 µL TBO, while (L+P−) and (L−P−) groups received 50 µL tryptone water. Plates were agitated for 1 min at 350 rpm, 35 °C using a PST-60 HL-4 thermoshaker (Biosan, Riga, Latvia). After incubation, irradiation was carried out under darkroom conditions in a Class II laminar flow hood (BIO ACTIVA VE 120, AQUARIA SRL, Lacchiarella, Italy), with the laser tip fixed 1 mm above sample surfaces; adjacent wells were shielded with matte black covers. Post-irradiation, 10 µL from each well was diluted in 4 mL tryptone water, vortexed, and 10 µL aliquots were plated in duplicate on Sabouraud agar (*Candida* spp.) or Columbia agar with 5% sheep blood (*S. aureus*). Cultures were incubated at 35 °C for 48 h (*Candida* spp.) or 37 °C for 24 h (*S. aureus*). Colonies were then quantified using a ProtoCOL 3 automated counter (Synbiosis, Cambridge, UK), and CFU/mL was calculated to determine the antimicrobial effect of combined photosensitizer and laser treatment.

### 2.4. Second Phase of the Study

A total of 1280 tests were conducted (320 for each tested microorganism), divided into four experimental groups outlined in Section 2.3.

In this phase, the effect of different laser settings (output power and irradiation time) was evaluated at the optimal TBO incubation time previously established for each microorganism. The outcome was expressed as a reduction in viable cells (CFU/mL). Sterile black 96-well microtiter plates with lids (Thermo Fisher Scientific, Waltham, MA, USA) were used, each well containing 200 µL of microbial suspension. To avoid cross-light diffusion, only 24 non-adjacent wells were used per plate. Wells in the (L+P+) and (L−P+) groups received 50 µL of TBO, while (L+P−) and (L−P−) wells received 50 µL of tryptone water. Plates were shaken in a PST-60 HL-4 thermoshaker (Biosan, Riga, Latvia) for 1 min at 350 rpm and 35 °C, then irradiated in a Class II laminar flow hood (BIO ACTIVA VE 120, AQUARIA SRL, Lacchiarella, Italy) under dark conditions at room temperature. In the (L+P+) and (L+P−) groups, irradiation was performed after the assigned incubation period, with the laser tip fixed 1 mm above the well surface. Adjacent wells were shielded with matte black covers. Following irradiation, 10 µL from each well was diluted in 4 mL tryptone water, mixed, and 10 µL aliquots were plated in duplicate on Sabouraud agar (*Candida* spp.) or Columbia agar with 5% sheep blood (*S. aureus*). Plates were incubated at 35 °C for 48 h (*Candida* spp.) or 37 °C for 24 h (*S. aureus*). Colonies were counted with a ProtoCOL 3 automatic counter (Synbiosis, Cambridge, UK), and CFU/mL values were calculated to determine microbial reduction resulting from the combined photosensitizer and laser treatment.

### 2.5. Statistical Analysis

Statistical analysis was performed using Statistica software, version 13.3 (StatSoft, Kraków, Poland). The initial phase in the statistical analysis was to establish the normal distribution of the data by means of the Shapiro–Wilk test. Subsequently, the Levene test was employed to ascertain the homogeneity of variances. The NIR test (Least Significant Difference [LSD] post hoc test) was deployed for comparative analyses between the study groups following ANOVA. Statistical significance was accepted at the *p* < 0.05 level.

## 3. Results

### 3.1. Phase I-Effect of Incubation Time

Incubation time with TBO significantly influenced the antimicrobial effect of aPDT across all tested microorganisms (Figure S1). The combination of photosensitizer and laser (L+P+) consistently reduced CFU counts compared with controls (L−P−) and with single-treatment groups (L−P+, L+P−), where no significant reductions were observed. For *S. aureus*, a strong reduction in viability was achieved after only 5 min of incubation (89.2%, *p* = 0.000001), whereas *Candida* strains generally required longer exposure. At 10 min, the reduction reached 85.9% for *C. albicans* (*p* = 0.000001), 86.9% for *C. krusei* (*p* = 0.010371), and 71.2% for *C. glabrata* (*p* = 0.004481). A mixed suspension of *S. aureus* and *C. albicans* also responded best at 10 min (87.7%, *p* = 0.000001). Prolonged incubation (15–30 min) did not consistently improve efficacy and, in some cases, was associated with a slight decline, suggesting possible diffusion effects or loss of effective photosensitizer concentration. These findings indicate that the minimal effective incubation times are 5 min for *S. aureus* and 10 min for *Candida* spp.

### 3.2. Phase 2-Effect of Laser Parameters

The efficacy of aPDT increased with higher power and longer irradiation times (Table 1). Across all strains, the strongest reductions were obtained at 400 mW for 120 s, which produced reductions of 99.0% for *S. aureus*, 95.2% for *C. albicans*, 90.1% for *C. krusei*, and 82.2% for *C. glabrata* (all *p* < 0.001). In the mixed suspension of *S. aureus* and *C. albicans*, microbial reduction reached 97.5% (*p* = 0.000001). At lower intensities, effects were less pronounced. For example, *C. albicans* showed only a 12.8% reduction at 100 mW for 30 s, while *C. glabrata* exhibited no significant effect under 100 mW for 30 s. Among the tested organisms, *S. aureus* was the most susceptible, showing significant reductions under nearly all tested settings, whereas *C. glabrata* was consistently the least responsive. Neither TBO alone nor laser irradiation alone produced meaningful reductions in CFU counts.

In Phase II, we evaluated the effect of laser parameters (irradiation time, output power) while keeping TBO incubation constant. aPDT efficacy varied with laser settings, underscoring the need for parameter optimization to ensure therapeutic effectiveness and safety. *S. aureus* ATCC 25923 showed significant CFU reduction across all aPDT groups. *C. albicans* ATCC 10231 was significant except at 300 mW/60 s, 400 mW/30 s, and 400 mW/60 s. For all microorganisms, significant reductions were observed with 200–400 mW and 30–120 s exposures, except for *C. albicans* under the conditions above and *C. glabrata* at 200 mW for 90–120 s.

No significant reductions were observed in control groups (L−P−, L+P−, L−P+). In the treatment group (L+P+), aPDT was most effective at higher power and longer exposure, with reductions exceeding 98% for *S. aureus*, 95% for *C. albicans*, 90% for *C. krusei*, and 82% for *C. glabrata* at 400 mW for 120 s (all *p* ≤ 0.000126). The dual suspension (*S. aureus* + *C. albicans*) also showed a strong reduction (97.5%) under the same conditions. Lowest reductions occurred at minimal settings (50–100 mW, 20–30 s), ranging from ~11–54%, often without statistical significance. Laser alone (L+P−) produced only minor reductions, ranging from ~3–21% across all strains, with most values not statistically significant. The highest effects were seen in *C. krusei* (20.9%, *p* = 0.0029) and the dual suspension (18.0%, *p* = 0.0128), while *S. aureus* showed ≤14% reduction. TBO alone (L−P+) also had minimal impact (2–12%), with no significant reductions observed in any strain. Overall, neither laser nor TBO alone demonstrated meaningful antimicrobial activity.

### 3.3. Key Findings

- Minimal effective incubation times: 5 min for *S. aureus*; 10 min for *Candida* spp.
- Most effective tested laser settings: 400 mW for 120 s, though these represent the upper limits of the tested range rather than confirmed optima.
- Relative susceptibility: *S. aureus* > *C. albicans* ≈ *C. krusei* > *C. glabrata.*
- Controls: Neither photosensitizer nor laser alone significantly reduced microbial counts.
- Levels of statistical significance were graphically presented in the figures. It should be noted, however, that the laser device used in this study had a maximum output of 400 mW, and while irradiation time could be extended beyond 120 s, this was not explored here. This represents a limitation of the present work.

## 4. Discussion

### 4.1. Importance of Incubation Time

Optimization of incubation time with TBO before light exposure is a critical determinant of aPDT effectiveness. The incubation period directly governs the penetration of the photosensitizer into microbial cells, its subcellular distribution, and the subsequent generation of ROS upon light activation. In our study, a statistically significant reduction in microbial viability was consistently observed after at least 10 min of TBO incubation for *Candida* spp. and 5 min for *Staphylococcus aureus*. These durations can be regarded as the minimal effective incubation times required for detectable antimicrobial action under the tested laser parameters. Notably, extending incubation beyond these thresholds (15–30 min) did not consistently produce superior reductions in CFU counts, and in some cases, the efficacy plateaued or slightly decreased. This observation suggests that overly prolonged incubation may cause diffusion of the photosensitizer away from the microbial surface, reducing its local concentration and limiting the oxidative damage upon irradiation. Conversely, very short incubation times may result in insufficient photosensitizer uptake, which compromises cytotoxic efficacy.

### 4.2. Comparison with Previous Studies on Incubation Time

Our findings broadly align with previous research, though differences in reported incubation requirements emphasize the complexity of standardizing protocols. Wiench et al. evaluated TBO-mediated aPDT under fixed laser parameters (P = 400 mW, t = 30 s) and concluded that 10 min was most effective for *C. albicans* ATCC 10231, though they reported lower reductions (76.89%) compared to our study (85.86%) [20]. For other strains, they observed maximum reductions at shorter incubation periods of 7 min, including *C. krusei* ATCC 6258 (59.39%), *C. glabrata* ATCC 90030 (61.36%), and *C. parapsilosis* ATCC 90018 (46.81%) [20]. Similarly, Chien et al. investigated fluconazole-resistant *C. albicans* strains and observed that 10 min of TBO incubation yielded maximal inactivation under a fluence of 50 J/cm^2^, while extending incubation to 30 or 60 min did not improve efficacy [29]. In contrast, Jackson et al. reported shorter optimal incubation times (5 min) for drug-resistant *C. albicans* isolates exposed to 21 J/cm^2^; longer exposure reduced cytotoxicity [30]. Donnelly et al., however, found 30 min to be the most effective for *C. albicans* NCYC 1467, with significantly diminished effects at shorter durations [31]. These conflicting results underscore that optimal incubation is strain- and condition-specific. Zhang et al. further challenged the concept of incubation dependence, reporting no correlation between incubation time (1, 5, or 10 min) and biofilm inactivation of *C. albicans* SC5314 and *C. tropicalis* ATCC 750. Instead, they observed that irradiation parameters, particularly combinations of higher power outputs (500–750 mW) and longer exposure times (1–2 min), were decisive in determining efficacy (*p* < 0.01) [32].

### 4.3. Broader Context of Incubation Parameters

Other studies illustrate the variability of incubation requirements across microbial species and experimental models. Nielsen et al. compared riboflavin and TBO as photosensitizers for aPDT against diverse microorganisms, including *C. albicans* ATCC 11775, *Enterococcus faecalis* DSM 20478, *Escherichia coli* ATCC 11775, *Lactobacillus paracasei* DSM 5622, *Porphyromonas gingivalis* ATCC 33277, *Prevotella intermedia* CCUG 24041, and *Cutibacterium acnes* DSM 1897. Using only 60 s of TBO incubation at 400 mW for 60 s (fluence 37.7 J/cm^2^), they achieved complete eradication of all tested organisms, with significantly greater efficacy than riboflavin (*p* < 0.001), except for *P. intermediaand*, *P. gingivalis*, where riboflavin also proved effective [33]. Moore et al. studied *S. aureus* ATCC 6538 and *Pseudomonas aeruginosa* PAO1 under conditions of 10 min TBO incubation, low power density (9.28 mW/cm^2^), and prolonged irradiation (60 min). They observed a 99.99% reduction in *S. aureus* and comparable efficacy for *P. aeruginosa* after 30 min. Interestingly, Gram-negative *P. aeruginosa* was more susceptible than Gram-positive *S. aureus*, likely due to the higher net negative charge of Gram-negative cell surfaces, which enhances the electrostatic attraction of the cationic photosensitizer [34,35,36,37,38,39]. These finding highlights that microbial cell wall structure plays a decisive role in photosensitizer uptake and sensitivity to aPDT, providing important insight for tailoring incubation strategies.

### 4.4. Influence of Laser Parameters

The second major determinant of aPDT efficacy is the light source. Our results demonstrated a clear dose–response relationship, with stronger reductions achieved at higher power outputs and longer irradiation times. The most pronounced antimicrobial effects were obtained with 400 mW for 120 s, corresponding to the highest tested fluence (48 J/cm^2^). Under these conditions, microbial reductions reached 99% for *S. aureus*, 95% for *C. albicans*, 90% for *C. krusei*, and 82% for *C. glabrata*. While these parameters were the most effective within the tested range, they cannot be considered true optima, since higher powers and longer exposures were not evaluated. It is possible that even greater reductions could be achieved with further increases, though excessive energy delivery raises concerns about tissue safety. Thus, our findings should be interpreted as identifying the most effective tested parameters, not necessarily the optimal therapeutic settings.

### 4.5. Evidence from Other Studies on Irradiation

Other investigations confirm the pivotal role of irradiation parameters. Wiench et al. reported that fluences between 30 and 40 J/cm^2^ consistently yielded up to 80% efficacy against *Candida* spp., regardless of whether cells were planktonic or biofilm-associated [21]. Barbério et al. evaluated a protocol using 0.05 mg/mL TBO, short irradiation (60 s), and low energy density (18 J/cm^2^) delivered by red LED. They found a 65% reduction in planktonic *C. albicans* (*p* < 0.001) compared to controls, and suggested that shorter irradiation times and lower fluences may be preferable for pediatric use [40]. Rosseti et al. demonstrated that irradiation with 68 mW for 21.47 J/cm^2^ following 10 min of TBO incubation enhanced ROS production, increased membrane permeability, and disrupted *C. albicans* biofilm formation, with a maximal reduction of 62% [41]. They quantified ROS accumulation using 2′,7′-dichlorodihydrofluorescein diacetate fluorescence and observed enhanced phagocytic activity of macrophages, highlighting the dual antimicrobial and immunomodulatory impact of aPDT [42]. These findings suggest that irradiation not only drives microbial death but also alters host–pathogen interactions.

### 4.6. Relative Susceptibility of Different Microorganisms

Our results also reveal significant interspecies differences. *C. albicans* displayed the highest sensitivity to aPDT, consistent with prior studies showing superior responsiveness of this species to TBO [43,44,45,46,47]. *C. glabrata*, by contrast, was the least susceptible. Wiench et al. attributed this to its ability to form multicellular aggregates that hinder photosensitizer penetration and reduce central bioavailability [13]. Our findings confirm this defensive strategy. Passos et al. observed similar resistance patterns in dual-species biofilms of *C. albicans* and *C. krusei*, where *C. albicans* was significantly inactivated but *C. krusei* was not [48]. Rodrigues et al. likewise reported that *C. krusei* exhibits relative resistance to oxidative stress, likely due to structural differences in cell wall or biofilm architecture [49]. These results underscore the need for species-specific adjustments in aPDT protocols and raise the possibility of combining photosensitizers or adjunctive agents to overcome resistance.

### 4.7. Clinical Implications

The rising global threat of antimicrobial resistance necessitates exploration of nontraditional therapeutic approaches [50]. Our findings support the clinical potential of TBO-mediated aPDT as either an adjunct to conventional therapies or, in selected cases, as a standalone modality. The tested protocol using FotoSan Agent, with 10 min incubation for *Candida* spp., 5 min for *S. aureus*, and 400 mW for 120 s, consistently achieved substantial pathogen reduction [51]. Importantly, the therapy also proved effective in mixed suspensions, indicating potential utility in polymicrobial oral infections where traditional pharmacotherapy often fails. Given its broad-spectrum activity and low risk of inducing microbial resistance, aPDT may become particularly valuable in immunocompromised patients or in cases of chronic or recurrent infections.

### 4.8. Limitations of the Study

Despite encouraging results, several limitations must be acknowledged. First, experiments were performed exclusively on planktonic cultures of reference strains, which do not replicate the complex architecture and resilience of clinical biofilms [52,53,54,55,56]. Second, environmental factors present in the oral cavity, including saliva, blood, and pus, may interfere with photosensitizer availability or absorb light, reducing efficacy. Third, while aPDT significantly reduced microbial counts, complete eradication was not achieved, and repeated treatments may be necessary to maintain antimicrobial effects. Fourth, TBO lacks specificity for pathogens, raising the risk of disrupting commensal microbiota and altering microbial homeostasis. Fifth, incubation times of 5–10 min may present practical challenges in clinical workflows, especially for routine applications. Finally, the cytotoxic impact of aPDT on host tissues was not assessed, and safety evaluations remain essential before clinical implementation [20,55].

### 4.9. Future Directions

Future research should focus on expanding experimental models to include clinical isolates, polymicrobial biofilms, and in vivo conditions that mimic the oral environment. Dose–response studies are needed to identify efficacy plateaus, safety thresholds, and optimal irradiation parameters. Combinatorial strategies, such as integrating aPDT with conventional antifungal or antibacterial agents, nanoparticles, or immunomodulatory therapies, may help overcome species-specific resistance. The development of user-friendly delivery systems that reduce incubation times and standardize dosimetry would further enhance clinical adoption. If validated in controlled clinical trials, TBO-mediated aPDT could provide a minimally invasive, resistance-independent, and broadly applicable antimicrobial strategy. This aligns with global priorities in combating antimicrobial resistance and offers promise for immunocompromised individuals and patients with persistent or recurrent infections [56,57].

## 5. Conclusions

This study demonstrated that TBO-mediated aPDT with a 635 nm diode laser effectively reduced *Candida* spp. and *Staphylococcus aureus* under in vitro conditions. The strongest effects were achieved with 10 min incubation for *Candida* and 5 min for *S. aureus*, followed by irradiation at 400 mW for 120 s. Antimicrobial efficacy required the combined use of both photosensitizer and light, as neither alone produced significant effects. These results support the potential of TBO-mediated aPDT as an adjunct to conventional therapy, with possible application as an alternative in cases of drug resistance. The identified conditions (≥400 mW, ≥120 s) provide a baseline for efficacy, though they should not be considered definitive optima, as higher doses were not tested. Further dose–response and clinical studies are needed to refine parameters and standardize therapeutic protocols.

## Figures and Tables

**Table 1:** Percentage reduction in viable cells (CFU/mL) in planktonic form for the photodynamic group (relative to the control group) under different laser parameters (output power; irradiation time). For each microorganism, gray shading indicates the highest efficacy of aPDT achieved under the applied parameters. (A) *Candida albicans* ATCC 10231; (B) *Candida krusei* ATCC 14243; (C) *Candida glabrata* ATCC 2001; (D) *Staphylococcus aureus* ATCC 25923; (E) mixed culture: *C. albicans* ATCC 10231 and *S. aureus* ATCC 25923.

Microorganism	Time (s)	50 mW	100 mW	200 mW	300 mW	400 mW
A. *Candida albicans* ATCC 10231						
	30	26.42%	12.78%	56.34%	66.42%	67.15%
	60	25.38%	38.69%	39.30%	77.23%	56.86%
	90	24.33%	37.88%	45.28%	76.03%	91.08%
	120	44.55%	68.85%	80.78%	92.88%	95.18%
B. *Candida krusei* ATCC 14243						
	30	32.91%	33.02%	60.63%	58.82%	66.46%
	60	31.63%	36.19%	51.25%	61.92%	72.62%
	90	26.26%	31.31%	62.63%	75.76%	72.22%
	120	35.35%	36.36%	56.06%	80.81%	90.05%
C. *Candida glabrata* ATCC 2001						
	30	19.80%	11.47%	41.08%	56.26%	55.92%
	60	12.12%	34.41%	40.48%	45.13%	47.57%
	90	15.13%	39.05%	47.54%	72.87%	71.83%
	120	21.02%	33.14%	41.65%	74.81%	82.23%
D. *Staphylococcus aureus* ATCC 25923						
	30	53.96%	77.48%	77.72%	76.60%	86.23%
	60	57.52%	76.15%	74.29%	88.71%	87.75%
	90	72.75%	81.14%	85.32%	98.75%	98.78%
	120	77.53%	84.40%	86.50%	98.78%	99.00%
E. Mixed culture (*C. albicans* ATCC 10231 + *S. aureus* ATCC 25923)						
	30	47.30%	60.70%	67.37%	71.68%	80.65%
	60	50.71%	62.49%	70.04%	82.30%	86.04%
	90	61.51%	65.95%	77.50%	94.68%	96.26%
	120	64.49%	68.97%	84.41%	97.14%	97.52%

## Data Availability

The original contributions presented in this study are included in the article/Supplementary Material. Further inquiries can be directed to the corresponding authors.

## Supplementary Materials

The following supporting information can be downloaded at: https://www.mdpi.com/article/10.3390/microorganisms13092126/s1, Figure S1. Influence of different incubation times (1–30 min) on the effect of photodynamic inactivation on reduction in viable cells (CFU/mL) in planktonic form. CFU/mL was determined after TBO-mediated aPDT (L+P+), treatment with light alone (L+P−), or treatment with the photosensitizer alone (L−P+), and compared to the negative control (L−P−). Data represent mean values ± standard deviations from four replicate experiments and indicate the comparative effect of aPDT versus the other treatment groups. Statistical significance levels are marked as follows: *** *p* < 0.001, ** *p* < 0.01, * *p* < 0.05. (A) Candida albicans ATCC 10231; (B) Candida krusei ATCC 14243; (C) Candida glabrata ATCC 2001; (D) *Staphylococcus aureus* ATCC 25923; (E) mixed culture: C. albicans ATCC 10231 and S. aureus ATCC 25923.
