# Supplementary material for: Assessment of the Impact of Antimicrobial Photodynamic Therapy Using a 635 nm Diode Laser and Toluidine Blue on the Susceptibility of Selected Strains of Candida and Staphylococcus aureus: An In Vitro Study
Source: Microorganisms. 2025 Sep 11;13(9):2126. doi: 10.3390/microorganisms13092126 (PMC12472698; doi:10.3390/microorganisms13092126)
Supplement: Supplementary file 1 [file microorganisms-13-02126-s001.zip › microorganisms-3782250-supplementary.pdf]

microorganisms

MDPI

Supplementary Materials

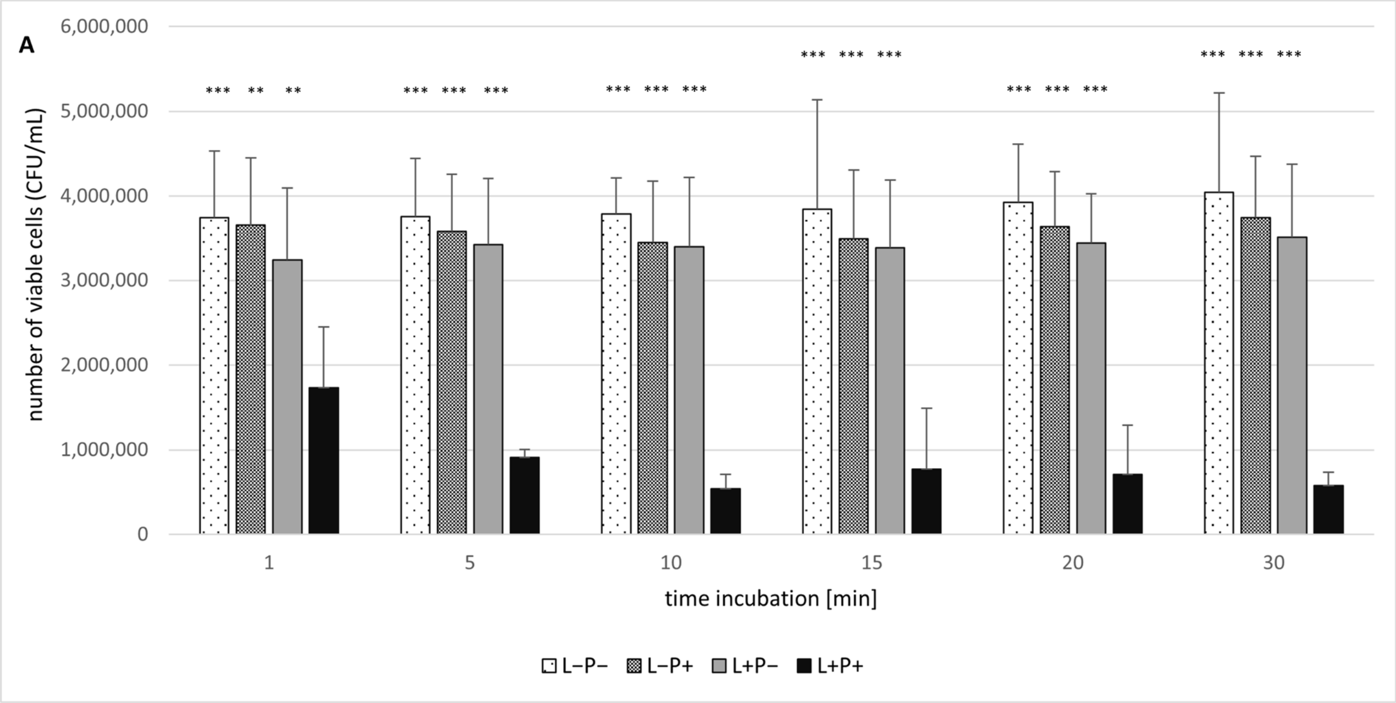

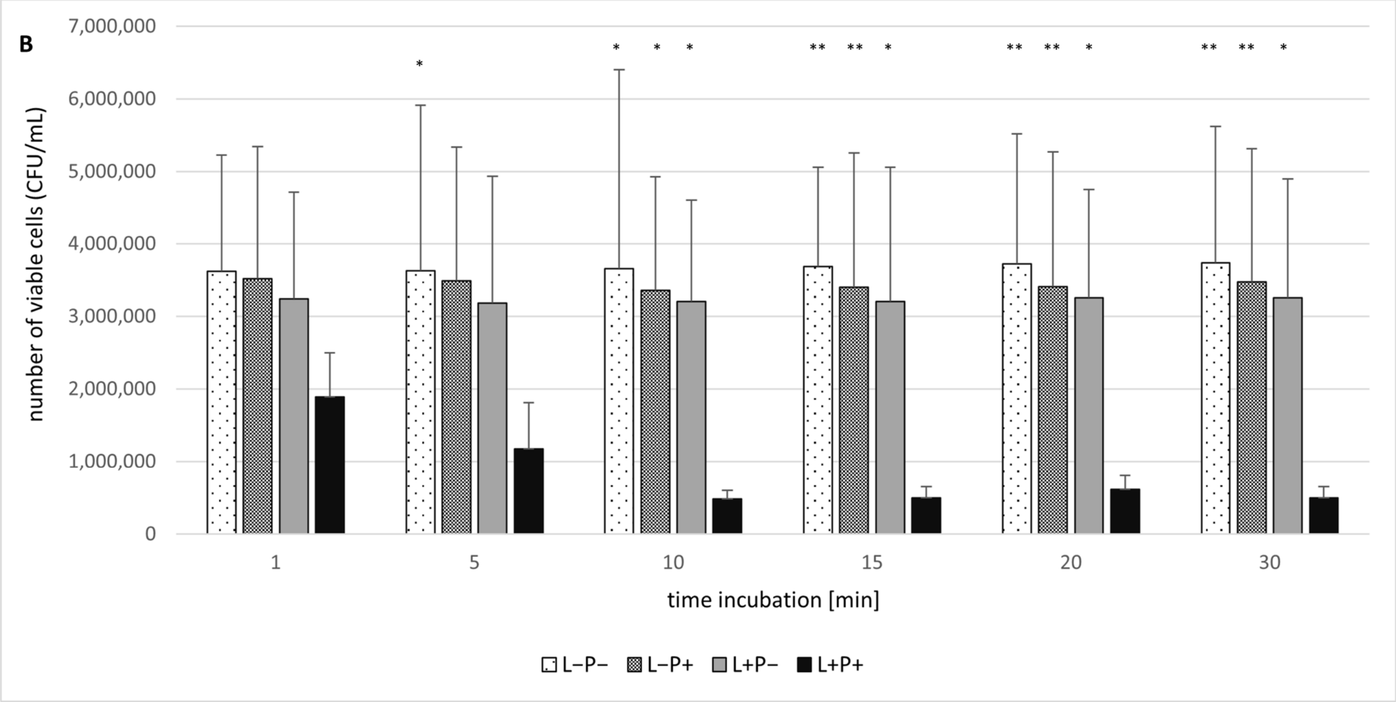

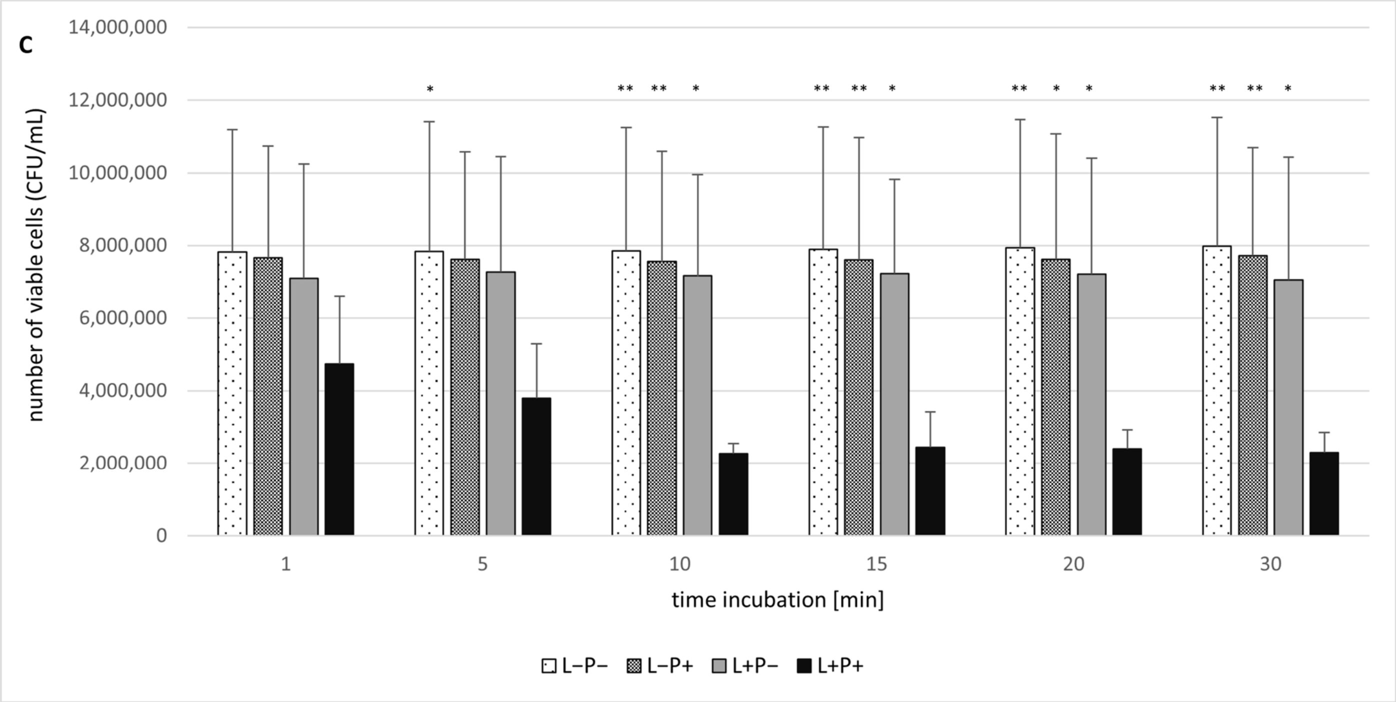
C
14,000,000
12,000,000
10,000,000
8,000,000
6,000,000
4,000,000
2,000,000
0
number of viable cells (CFU/mL)
1
5
10
15
20
30
time incubation [min]
L−P−
L−P+
L+P−
L+P+

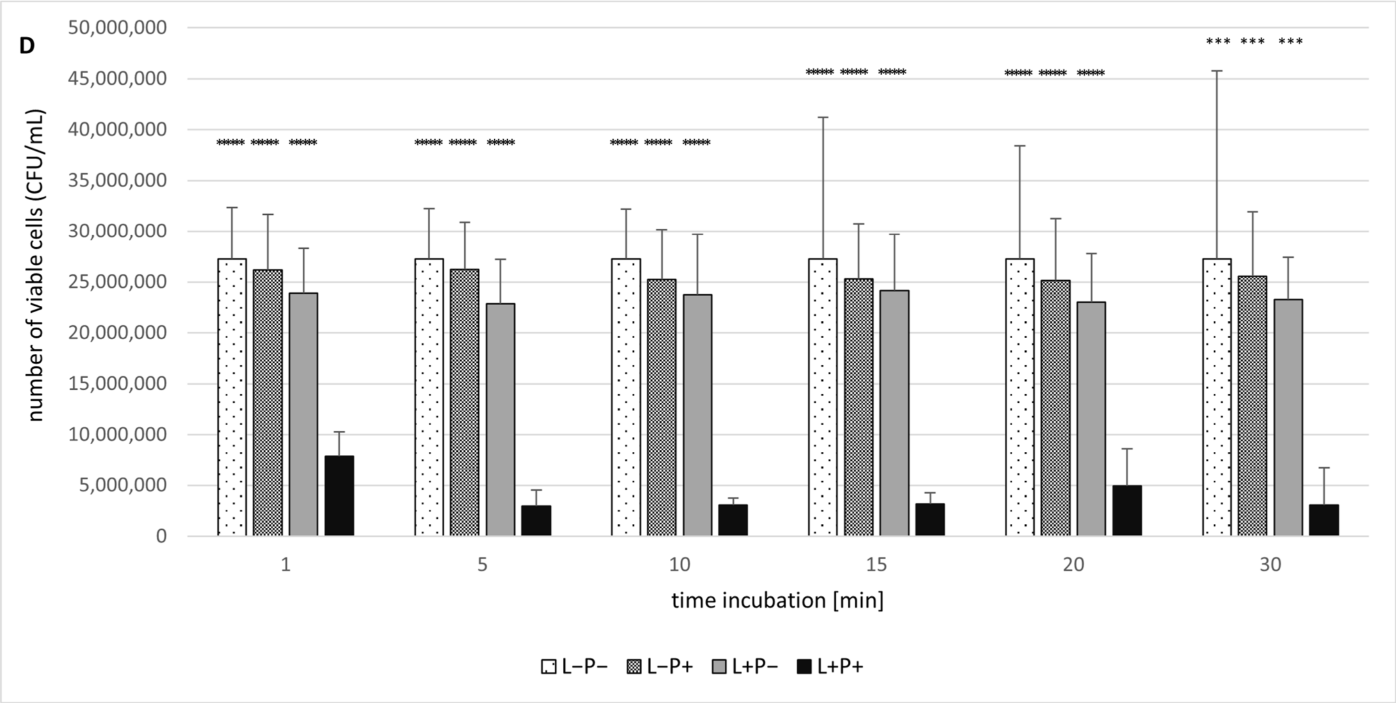
D
50,000,000
45,000,000
40,000,000
35,000,000
30,000,000
25,000,000
20,000,000
15,000,000
10,000,000
5,000,000
0
number of viable cells (CFU/mL)
1
5
10
15
20
30
time incubation [min]
L−P−
L−P+
L+P−
L+P+

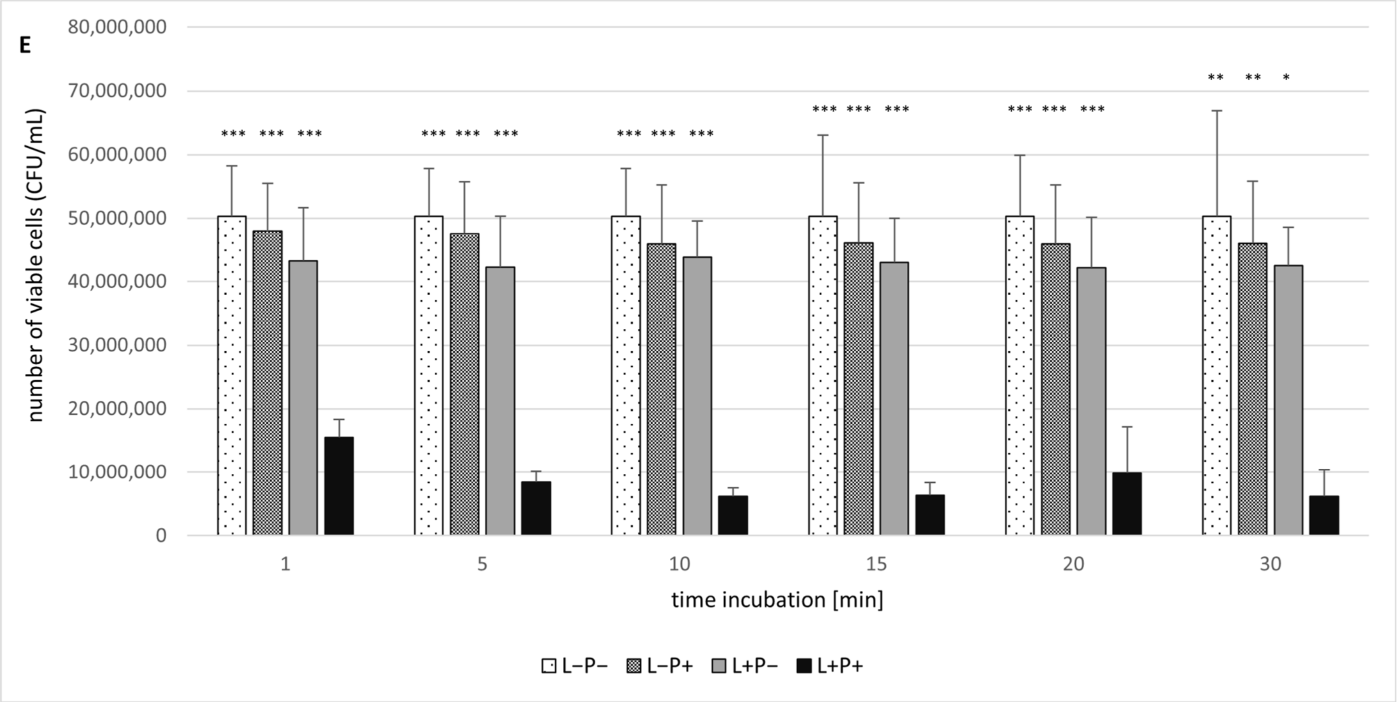

Figure S1. Influence of different incubation times (1–30 min) on the effect of photodynamic inactivation in reduction of viable cells (CFU/mL) in planktonic form. CFU/mL was determined after TBO-mediated aPDT (L+P+), treatment with light alone (L+P–), or treatment with the photosensitizer alone (L–P+), and compared to the negative control (L–P–). Data represent mean values ± standard deviations from four replicate experiments and indicate the comparative effect of aPDT versus the other treatment groups. Statistical significance levels are marked as follows: ***$p < 0.001$, **$p < 0.01$, *$p < 0.05$. (A) *Candida albicans* ATCC 10231; (B) *Candida krusei* ATCC 14243; (C) *Candida glabrata* ATCC 2001; (D) *Staphylococcus aureus* ATCC 25923; (E) mixed culture: *C. albicans* ATCC 10231 and *S. aureus* ATCC 25923.
